# A simple method for evaluation pharmacokinetics of glycyrrhetinic acid and potential drug-drug interaction between herbal ingredients

**DOI:** 10.1038/s41598-019-47880-4

**Published:** 2019-08-05

**Authors:** Neng Zhou, Caiyuan Zou, Menglin Qin, Yi Li, Jiayi Huang

**Affiliations:** Guangxi Key Laboratory for Agricultural Resources Chemistry and Biotechnology, Colleges and Universities Key Laboratory for Efficient Use of Agricultural Resources in the Southeast of Guangxi, College of Chemistry and Food Science, Yulin Normal University, Yulin, 53700 China

**Keywords:** Molecular medicine, Chemical biology

## Abstract

A simple validated high performance liquid chromatography method was developed for the evaluation of the effect of three kinds of active ingredients in traditional Chinese medicine (TCM) on the pharmacokinetics of glycyrrhetinic acid (GA),a kind of active component from the most commonly used TCM licorice. Our results revealed that all of the calibration curves displayed good linearity. Intra- and inter-day precision for GA ranged from 2.54 to 3.98% and from 4.95 to 7.08%, respectively. The recovery rates for GA were determined to be 96.3–106.4%. All the samples showed satisfactory precision and accuracy in various stability tests. Plasma pharmacokinetic parameters including area under the concentration-time curve (AUC), elimination half-life (t_1/2_), time to peak concentration(T_max_) and peak concentration C_max_ were calculated. No significant difference was found as compared the groups administrating GA with and without other ingredients from TCM.

## Introduction

**Figure 1:**
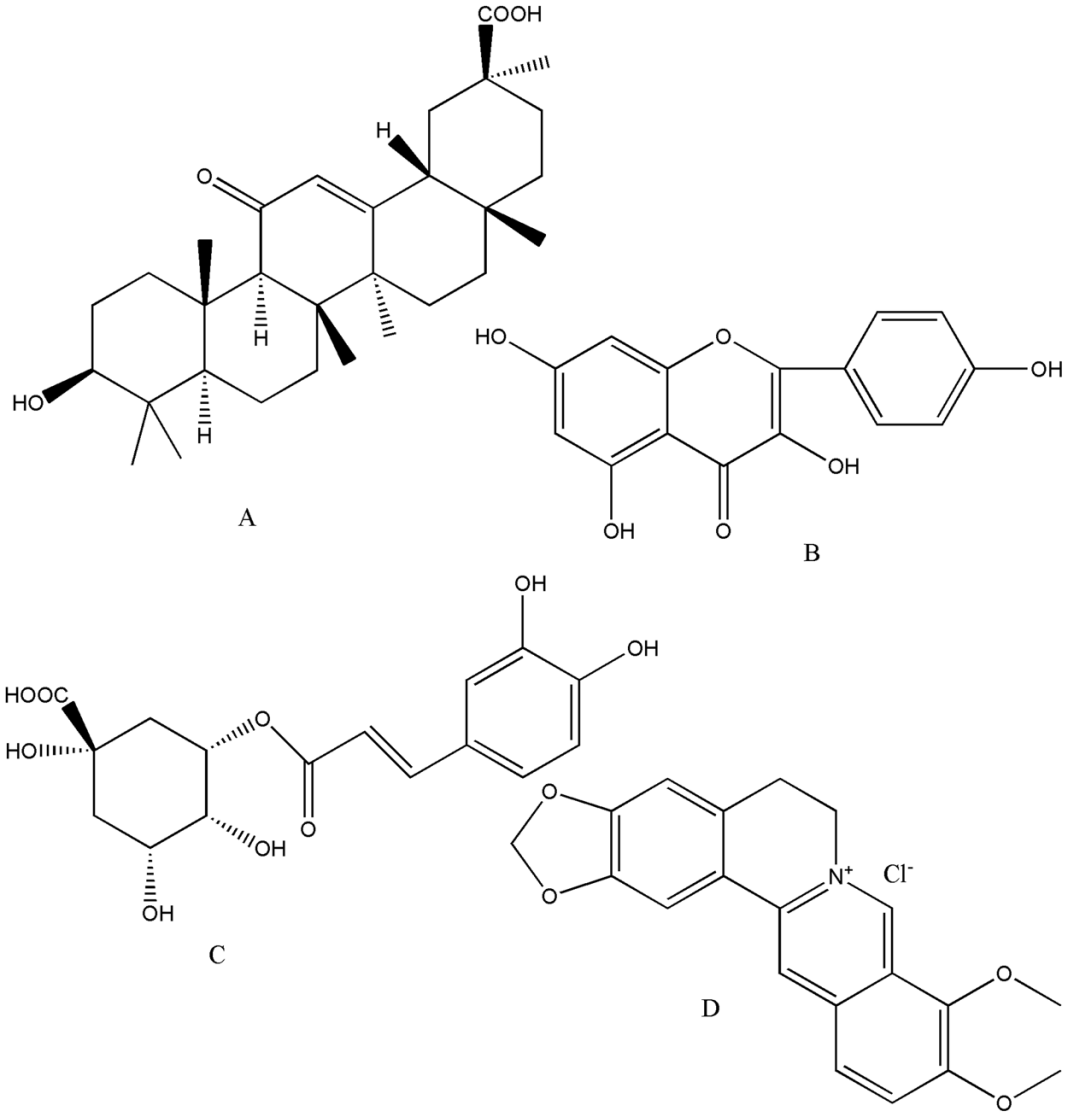
Structure of GA, KL, CA and BB (from **A**–**D**).

Licorice is the most commonly used Chinese medicine in prescription^1,2^, which is mainly used to treat peptic ulcer, cough, and hepatitis C^3^. Glycyrrhizic acid is one of the characteristic ingredients of licorice. When administrated Chinese medicine containing licorice, glycyrrhetinic acid (GA, see Fig. 1 for its structure), namely metabolite of glycyrrhizic acid, is the mainly detected form of it in plasma. For GA, on the one hand pharmacological activities such as anti-inflammatory^4,5^, antivirus^6^, hepatotoxin protection^7^, antiulcer^8^, antitumor^9,10^ and adrenal cortical hormone kind function^11^ have been reported. On the other hand, GA is reported to be an inhibitor of P-glycoprotein and multidrug resistance protein 1^12^. So GA may play a synergistic role with other ingredients in prescription of TCMs during treatment through inhibiting the efflux of these components so as to enhance their efficacy. Some studies have provided supports to it. For example, glycyrrhizin and licorice significantly affect the pharmacokinetics of methotrexate in rats could be explained by the higher serum level of GA^13^. The liquorice promoted the absorption of peoniflorin in peony and enhanced its concentration^14^. Glycyrrhizic acid increased AUC of some alkaloids^15^.

However, as an active component which have clearly shown to be an effective agent for treatment of atopic dermatitis^16^ and purulent scar disease^17^, few data are available to address the effects of active ingredients of traditional Chinese medicine (TCM) on the pharmacokinetics of GA. Moreover GA usually administrated in form of mixture such as formula of TCM, in which exist many other ingredients. So the present studies design a procedure to focus on the effect of oral administration of three kinds of ingredients from TCM on the plasma of GA in an animal model. These ingredients include kaempferol (KL), chlorogenic acid (CA) and berberine (BB) (see Fig. 1 for their structure). Though some HPLC-MS/MS^18–21^ or UPLC–MS/MS^22,23^ methods have been reported for the determination of GA. However that mean more expensive instrument and high operation and maintenance fee are needed. Thus, a simple validated high performance liquid chromatography method was developed here for the evaluation of these effects.

## Experimental

### Chemicals and animals

Glycyrrhetinic acid (GA) was a product of Spain obtained from Fluka. Kaempferol (KL), chlorogenic acid (CA) and berberine (BB) were obtained from Shanghai Aladdin Biochemical Technology Co., Ltd (China). Methanol (HPLC grade) was obtained from Tianjin Si You fine chemicals company (China). All other chemicals were of analytical grade. Water was distilled water.

Sprague-Dawley (SD, specific pathogen free level) rats (230 ± 50 g),male and female each half, were purchased from Experimental Animal Center of Guangxi Medical University (Guangxi, China). They were kept under room temperature for 2 days before study.

### HPLC conditions

Chromatography analysis was carried out on a CTO-20A type Shimadzu high performance liquid chromatography (HPLC) with SPD-20A and LC-20AT unit, CTO-20AC column oven and a Shimadzu C18 column (4.6 × 250 mm). The mobile phase was delivered at a 1.0 mL/min flow rate using isocratic elution. Mobile phase is consisted of methanol-0.4% phosphoric acid (85:15) and was passed 0.45 μm membrane before use. The injection volume was 20 μL. The column temperature was set at 35 °C and the wavelength used was at 251 nm.

### Preparation of solutions

GA Stock solutions: Stock solutions for HPLC method was prepared by dissolving GA in methanol to obtained 500 ng/mL. The solution was then diluted with methanol to achieve standard working solutions.

Anticoagulant: 1.20 g ammonium oxalate and 0.80 g potassium oxalate dissolved in 100 mL water. Sodium carboxymethyl cellulose (SCC): Put 1.25 g SCC in Beaker and ultrasonically dissolve in 50 mL water then add water to 250 mL to make 0.5% solution.

GA gavage solution: prepared in 0.5% SCC solution with or without KL, CA or BB.

Quality control (QC) samples: 100 μL three level concentration of GA were transferred to centrifuge tube, dried by N_2_ flow under a 50 °C water bath and 100 μL blank plasma was added. And then perform according to the sample preparation procedure.

### Sample preparation

The frozen plasma samples were thawed under room temperature, mix well with a pipette. Then 100 μL was transferred to a 2 mL centrifuge tube, add 1 mL acetonitrile and then extract 3 min by vortex. Then centrifuge 8 min at a speed of 12000 r/min. 1 mL of the supernatant was transferred to another centrifuge tube, dried by N_2_ flow under a 50 °C water bath. The dried samples were re-dissolved in 100 µL methanol. After vortex 1 min, they were centrifuged 5 min at a speed of 12000 r/min. 20 µL of the supernatant was directly sampled in HPLC analysis.

The frozen tissues were naturally thawed. Then cut into several large pieces with scissors, carefully remove impurities such as blood clots, etc., and then cut into small one, dry with filter paper. 0.20 g of these tissues was put into a centrifuge tube. Then the stuff was homogenized with a homogenizer by addition of 0.5 mL physiological saline. 2 mL acetonitrile was added in succession and extract 1 min by vortex. Then centrifuged 5 min at a speed of 12000 r/min.2 mL of the supernatant was transferred to another centrifuge tube, dried by N_2_ flow under a 50 °C water bath. The dried samples were re-dissolved in 100 µL methanol. After vortex 1 min, they were centrifuged 5 min at a speed of 12000 r/ min. 20 µL of the supernatant was directly sampled in HPLC analysis.

## Method Validation

### Selectivity and specificity

The selectivity and specificity of the method was investigated by screening analysis of rat blank and genuine plasma or tissue samples. The chromatogram of each blank and genuine sample was compared with those spiked with GA to ensure no peak interference.

### Linearity and sensitivity

The linearity of the method was evaluated by preparing five different concentrations of samples in plasma or tissue using the same extraction procedure as sample preparation. The calibration curves were got by plotting peak area of GA to plasma (µg/mL) or tissue (µg/g) concentration using a linear least-squares regression model. The lower limit of detection (LOD) was the concentration giving a signal-to-noise ratio at least 3-fold. The lower limit of quantity (LOQ) was the concentration giving a signal-to-noise ratio at least 10-fold.

### Precision and accuracy

The within-day and between-day precision and accuracy were investigated by determining QC samples at three different concentrations (three replicates for each concentration level) over three consecutive days. The samples concentrations were calculated using calibration curves obtained. The precision of the method was expressed as the relative standard deviation (RSD) and the accuracy was described as recovery (RE). The suitability of the precision and accuracy was assessed by the following criteria: the RSD should not exceed 15% and the accuracy should be within 20% of the actual values for QC samples^18^.

### Extraction recovery

The extraction recovery of GA in QC samples at three levels was performed by calculating as the ratio of the peak area of extracted QC samples to that of the equivalent concentration of standard GA solution.

### Sample stability

Samples stability of repeated freezing and thawing, 4 hours placement at room temperature(4 h at RT), 8 hour placement after extraction(8 h after EX) and frozen at −20 degrees Celsius for 2 weeks were investigation using QC samples at three concentration levels.

### Pharmacokinetics and liver tissue distribution study

The animal study protocol was approved by the Ethical Committee of College of Biology & Pharmacy of Yulin Normal University (Yulin, China). 54 SD rats were randomly divided into 9 groups, each group of 6, male and female half. 9 groups were used in pharmacokinetics and liver issue distribution study of GA. After fasting 12 h (Free access to water), each rat was administered 50 mg/kg GA by gavage. Then 1 mL blood was taken from eye ball after 0.25, 0.5, 1.0, 1.5, 2.0, 3.0, 4.0, 6.0 and 8.0 h, respectively. For evaluation of the effect of active ingredients of traditional Chinese medicine on plasma concentration and tissue distribution of GA, rat was administered 50 mg/kg GA with KL (250 and 500 mg/kg), CA (200 and 400 mg/kg) or BB (200 and 400 mg/kg) by gavage, respectively. Then 1 mL blood was taken from eye ball at specified time. Each time point uses 6 rats. The blood was put into a centrifuge tube in which anticoagulant was added into beforehand. The plasma was made by centrifuging at 3000 r/min for 10 min. After blood was taken, the rats were sacrificed at once and the tissues were removed immediately. Then, the blood was removed from the surface of the tissue by washing with 0.9% saline, and the brine was dried with a clean filter paper. All samples obtained in this step were stored at −20 °C in a refrigerator until analysis.

### Data analysis

The pharmacokinetic parameters (C_max_, T_max_, AUC, MRT(mean residence time), t_1/2_, CL/F(apparent total body clearance rate)) of GA were calculated by noncompartmental analysis of plasma concentration vs time with DAS 3.3.0. The comparison between two groups was performed by SPSS 19.0 (Statistical Package for the Social Science) using a analysis of variance. The statistical significances of differences between the groups were compared and a value of p < 0.05 was considered statistically significant.

## Results

### Selectivity of HPLC analysis

**Figure 2:**
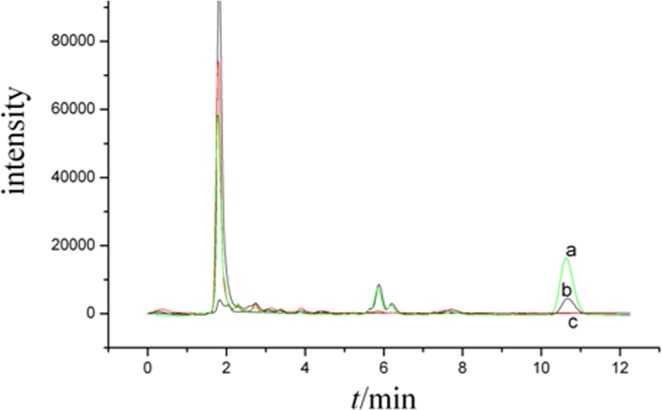
Chromatogram of plasma samples of GA blank plasma spiked with 15.0 μg/mL GA; b. plasma sample 3.5 h after oral administration GA; c. blank plasma.

The typical chromatograms of plasma were shown in Fig. 2. The retention time of BB is at about 10.67 min. The chromatograms were free of interfering peaks from endogenetic substance at the retention time of GA. The retention time of GA in liver is about 11.5 min. Also, none of interfering peaks from endogenetic substance at the retention time of GA are found. KL,CA and BB did not interfere the determination of GA due to the difference of their polarities.

### Standard curves

Respectively, a volume of 100 uL of 0.5, 1.0, 5.0, 10.0, 15.0 μg/mL standard GA solutions were taken into a 2-mL centrifuge tube and dried by nitrogen flow. Take the same volume of rat blank plasma and add into these tubes in succession. After vortex mixing, a series of drug-containing plasma were made, which concentration was 0.1, 2.5, 5.0, 10.0, 15.0 μg/mL, respectively. According to the above-mentioned sample extraction method, a series of processes such as extraction, re-dissolving and measurement were done and the peak area was obtained. The average area of triplicate was obtained as the ordinate and the concentration of GA (μg/mL) was as the abscissa. The standard curve was got after linear regression as the following: y = 21229x-3466 (R^2^ = 0.9991). The result showed a good linear relation was obtained as the plasma concentration of GA was between 0.5–15.0 μg /mL. The LOD was 0.17 μg /mL and the LOQ was 0.19 μg /mL.

For tissue distribution, a volume of 100 uL of 0.5, 1.0, 5.0, 10.0, 15.0 μg/mL standard GA solutions were taken into a 2-mL centrifuge tube respectively and dried by nitrogen flow. 0.20 g tissue was added to make 0.25, 0.50, 0.75, 1.5 and 2.5 µg/g tissue samples. Then treat as the sample preparation procedure. The standard curve was got after linear regression as the following: y = 24051x-344 (R^2^ = 0.9956).

### Precision and accuracy

The precision and accuracy of the method were studied by determining QC samples at three different concentrations (three replicates for each concentration level) over three consecutive days. The precision of the method at each QC concentration was expressed as the relative standard deviation (RSD) and the accuracy was described as recovery. The results are presented in Table 1. As can been seen from Table 1, the precisions of the three kinds of samples in three concentration level are lower than 8% (RSD) for within-day and between-day precision, respectively. The precision and accuracy can meet the need of the study.

**Table 1:** Results of precision and accuracy(n = 3).

	Concentration(μg/mL(g))	within-day precision RSD/%	between-day precision RSD/%	Recovery/%
Plasma	0.5	3.86	6.92	97.3
	5.0	3.69	5.91	96.8
	15.0	3.28	7.08	99.0
Liver	0.25	3.98	5.22	105.2
	0.75	2.54	6.37	96.3
	2.5	3.53	4.95	106.4

### Sample stability

Stability of QC samples in tissues was investigated at three concentration levels under different storage conditions: long-term stability at −20 °C for 2 weeks, pre (post)-preparative stability at room temperature for 4 (8) h, and three freeze-thaw cycles. The stability of Samples was expressed as the relative standard deviation (RSD). The results could be found in Table 2. These results show the samples were stable during the study.

**Table 2:** Stability of GA in samples (n = 3).

Sample	concentration (μg/mL(g))	freeze-thaw cycle RSD/%	4 h at RT RSD/%	8 h after EX RSD/%	−20 °C for 2 weeks RSD%
Plasma	0.5	10.5	2.9	1.9	6.6
	5.0	4.1	1.2	5.5	5.0
	15.0	5.1	2.0	5.8	9.3
Liver	0.25	—	5.7	6.3	11.1
	0.75	—	4.3	4.9	9.8
	1.50	—	3.6	5.2	12.2

—: Not done.

### Extraction recovery

The extraction recovery of GA at three QC levels was conducted by calculating the ratio of the peak area of blank tissue spiked with GA and the peak area of the standard QC solution. In plasma, extraction recovery is 79.6%, 83.1%, 85.2% (average 81.6%) for three QC samples, respectively. In liver, extraction recovery is 62.7%, 66.0% and 69.5% (average 66.0%) for three QC samples, respectively.

## Results of Pharmacokinetics

### Plasma concentration of GA and its pharmacokinetics parameters

**Figure 3:**
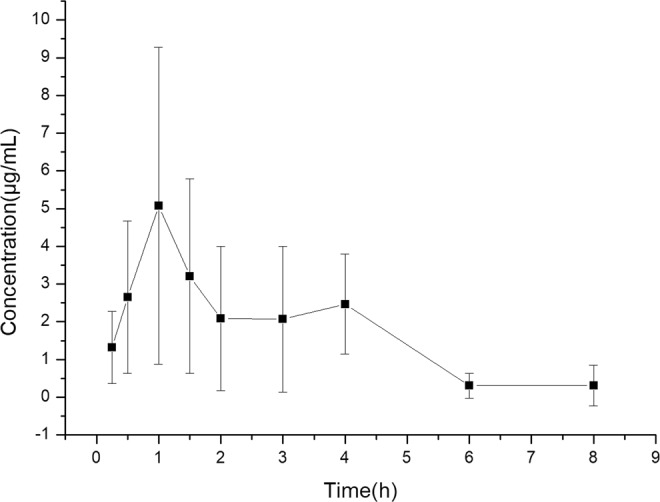
Mean plasma concentration-time results in rats after oral administration of 50 mg/kg GA.

The validated method was subsequently applied to determine the concentration of GA in plasma after oral administration at a dose of 50 mg/kg to rats. The mean plasma concentration-time profiles of GA in rats after oral administration was shown in Fig. 3. The pharmacokinetic parameters of GA were calculated by noncompartmental analysis of plasma concentration vs. time with DAS 3.3.0 software. AUC (0-t) and AUC (0-∞) were 13.73 ± 4.09 and 15.14 ± 5.79 mg/L.h, respectively. T_1/2_ and T_max_ were 1.49 ± 1.15 and 1.33 ± 0.41. C_max_, MRT(0-t) and CL/F were 6.41 ± 2.59 mg/L, 2.59 ± 0.54 h and 7.78 ± 4.03 L/h/kg, respectively.

### Liver concentration of GA

**Figure 4:**
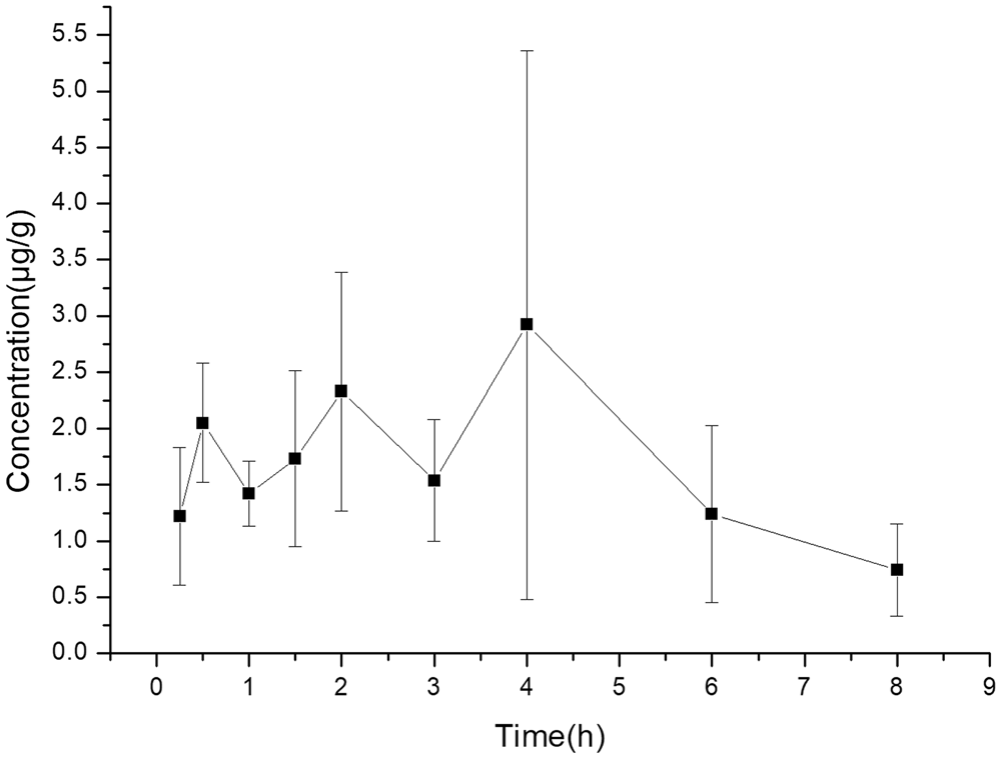
Mean liver concentration-time results in rats after oral administration of 50 mg/kg GA.

Using the validated method for liver, concentration of GA in it was determined after oral administration at a dose of 50 mg/kg to rats. The mean liver concentration-time profiles of GA in rats after oral administration was shown in Fig. 4. The maximum concentration in liver was at about 4 h.

### Effects of active ingredients from TCM on plasma concentration of GA

Effects of three kinds of active ingredients from TCM, namely Kaempferol (KL), chlorogenic acid (CA) and berberine (BB), on the plasma concentration of GA were investigated and the results were shown in Table 3. As can be seen from this table, KL and BB had significant effect on the plasma concentration of GA at some time points.

**Table 3:** Effects of active ingredients of TCM on plasma concentration of GA (µg/mL, n = 6).

mg/kg	0.5 h	1.0 h	2.0 h	3.0 h	4.0 h
GA	2.65 ± 2.02	5.08 ± 4.20	2.09 ± 1.91	2.07 ± 1.93	2.47 ± 1.33
GA + BB (200)	—	4.93 ± 3.21(0.9)	5.45 ± 3.08 (0.05)	—	3.03 ± 1.51 (0.3)
GA + BB (400)	—	4.79 ± 1.49 (0.9)	3.35 ± 1.80 (0.3)	—	3.29 ± 3.28 (0.5)
GA + CA (200)	2.85 ± 0.63(0.8)	2.37 ± 0.86 (0.2)	—	—	1.86 ± 0.77 (0.7)
GA + CA (400)	1.49 ± 0.24(0.2)	2.45 ± 0.71 (0.2)	—	—	2.34 ± 0.32 (0.9)
GA + KL (200)	—	3.67 ± 1.15 (0.4)	5.60 ± 2.47(0.02)	3.26 ± 2.44(0.2)	—
GA + KL (500)	—	4.36 ± 2.01 (0.7)	4.21 ± 3.34 (0.2)	3.30 ± 1.10(0.04)	—

—, not done; Data in parentheses are actual p-values, compared to GA group; p < 0.05 was considered statistically significant.

## Discussions

### Chromatographic determination

The present study devoted to develop a simple method for the determination of GA in plasma and liver, including HPLC condition and sample preparation. After testing, isometric elution instead of gradient elution was adopted. Considering a good peak shape, effective separation and less time be obtained, methanol-0.4% phosphoric acid was chosen as a mobile phase. As a deproteinized reagent, acetonitrile was more effective than methanol here. For liver, there are interference peak near the target peak. So we reconstituted the N_2_ dried samples twice. First, acetonitrile was applied to re-dissolve, and then it was dried by N_2_ flow after transferring to another tube. Second, 100 µL methanolwas used to re-dissolve. After vortex 1 min, they were centrifuged 5 min at a speed of 12000 r/min. 20 µL of the supernatant was directly sampled in HPLC analysis. In this case, a good result was achieved.

### Pharmacokinetics of GA

As a metabolite of glycyrrhizic acid (GL) which is one of the characteristic ingredients of licorice, hardly any data is available of to address the pharmacokinetics of GA after oral administration of it, especially the tissue distribution. What we learned about the pharmacokinetics of GA were that after oral administration of GL or licorice^19–24^, in which GL was hydrolyzed into GA by intestinal bacteria^25^. This was just because when GL or licorice was administrated, GL or GL in licorice was almost totally metabolized into GA^24^. Moreover, GA is a substance with many pharmacological activities as mentioned above^4–10^. Hence, we here designed this work to investigate the oral pharmacokinetics of GA. The present result is comparable with another research in which water suspensions of coarse GA was applied^26^. However, the obtained pharmacokinetics parameters here included not only plasma concentration but also the corresponding tissue distribution.

Licorice is a herbal medicine, also a traditional Chinese medicine. So it is usually used in form of prescription, in which exist many ingredients. Thus herb–herb and drug-drug interactions involved in licorice in TCM have attracted more and more attentions. Some studies started from prescription. As compared to administration of licorice alone, AUC of GA was found to be significant increased after licorice (*Glycyrrhizae radix*) combined with *Cinnamomi ramulus* (p < 0.01)^27,28^. Similar result has occurred while licorice (*Glycyrrhizae Radix*) combined with *Sophorae flavescentis radix*^29^ and with *paeoniae radix*^30^. Different proportions of other crude drugs with licorice in a prescription would lead to significant different pharmacokinetics of GA^31,32^. Of course, in some research, as compared to administration of licorice alone, AUC of GA was found to be significant decreased after licorice combined with other crude drugs, such as *Euphorbia kansui*^30^ and processed *Aconiti kusnezoffii radix*^33^. However, prescription is a complicate system. Which ingredient leads to significant difference of GA pharmacokinetics is unclear now. So component-component interaction has been designed to reveal this mystery. For example, paeoniflorin has been found to dramatically inhibited the absorption of glycyrrhizic acid in rats, but had only small effects on the absorption of GA after the administration of single glycyrrhizic acid or co-administration with paeoniflorin^34^. Another study discovered baicalin (a kind of flavone) significant decreased absorption of GA by *in situ* single-pass intestinal perfusion^35^. From the perspective of absorption mechanism, GA is reported to be a substrate^35^ and an inhibitor^36^ of P-glycoprotein. BB^37,38^ and KL^39,40^ are inhibitors and substrates of P-gp. In this case, there may be exist possibility of drug-drug interaction between GA and BB or KL. Therefore, in the present work, considering GA is an organic acid, we selected and evaluated the effects of Kaempferol (a kind of flavone), chlorogenic acid (a kind of organic acid like GA) and berberine (a kind of organic base) on the plasma concentration of GA. However, our results show no significant difference between the group with and without active ingredients of traditional Chinese medicine at the same time point. We speculate GA maybe a stronger substrate than ingredients we have studied here. Of course, more evidences are need.

## Conclusions

A simple validated high performance liquid chromatography method was developed for the evaluation of plasma concentration and tissue distribution of GA and the effect of three kinds of active ingredients in traditional Chinese medicine on it. The results would provide useful data for the application of glycyrrhetinic acid in clinical trials.

## Data Availability

The data generated and analysed in this study are available from the authors on request.
